# Clinical Experience Comparison of Foreign-Trained Dentists and Domestic Dental Students: One Institution’s Experience

**DOI:** 10.3390/dj12050139

**Published:** 2024-05-11

**Authors:** Zabihulla Ahmadi, Judy Chia-Chun Yuan, Michael Spector, Adriana Semprum-Clavier, Cortino Sukotjo, Fatemeh S. Afshari

**Affiliations:** 1 Department of Restorative Dentistry, College of Dentistry, University of Illinois Chicago, 801 S. Paulina St, Chicago, IL 60612, USA; zahmad2@uic.edu (Z.A.); yuanjudy@uic.edu (J.C.-C.Y.); 2 Department of Periodontics, College of Dentistry and Dental Clinics, University of Iowa, 801 Newton Rd, Iowa City, IA 52242, USA; michael-spector@uiowa.edu; 3 Advance Standing Dental Degree Program, Department of Restorative Dentistry, College of Dentistry, University of Illinois Chicago, 801 S. Paulina St, Chicago, IL 60612, USA; asemprum@uic.edu; 4 Predoctoral Implant Program, Department of Restorative Dentistry, College of Dentistry, University of Illinois Chicago, 801 S. Paulina St, Chicago, IL 60612, USA

**Keywords:** dental education, clinical competence, cultural diversity, foreign professional personnel/education, health professionals

## Abstract

This study compared the clinical experiences of foreign-trained dentists (FTDs) enrolled in an Advance Standing DMD Dental Program (DMDAS) with those of the domestic dental students (DMD) at the University of Illinois Chicago, College of Dentistry (UIC-COD). A cross-sectional retrospective chart review of patients treated by 295 DMD and 253 DMDAS predoctoral dental students was completed at the UIC-COD. The data were retrieved from the electronic health record system (axiUm) for the graduated classes of 2018, 2019, 2020, 2021, and 2022 on various performed clinical procedures as measured by relative value units (RVUs). The retrieved data were used to compare the clinical experiences of DMDAS vs. DMD students. Descriptive (mean) and statistical (independent t-test) analyses were used (α = 0.05). The results indicated that DMD and DMDAS students had comparable clinical experiences in several disciplines, including diagnosis, prevention, direct/indirect restorations, endodontics, periodontics, complete dentures, removable partial dentures, implants/fixed partial dentures, and oral surgery. There was a statistical difference in total RVUs for diagnosis (*p* = 0.002) and direct restorations (*p* < 0.001), in which DMD students had more experience. The 28 month program for FTDs appeared to be a reasonable timeframe to obtain an adequate number of varied clinical experiences as compared with the traditional four-year program at the UIC-COD.

## 1. Introduction

Healthcare professionals from across the globe with diverse backgrounds and training come to the United States (U.S.) in hopes of furthering their education and ultimately seeking employment in this country. These hopefuls include foreign-trained dentists (FTDs). Dental institutions began enrolling foreign-trained dentists in modified predoctoral programs as early as the 1970s [1]. Many different pathways exist for the FTDs to matriculate in U.S. dental institutions [2,3], including enrollment in an advanced program or international dental programs (IDPs). Currently, 41 of 71 dental schools offer an International Dental Program (IDP) to FTDs. Approximately 600 FTDs matriculate into IDPs in the U.S. annually [4]. According to the Institute of International Education, the U.S. has been the main destination for most of these foreign-trained students seeking to further their education and research [5]. Based on a self-reported study, the majority of FTDs have improved their knowledge and clinical skills after completion of their programs [6]. Studies on the topic of FTDs mainly focus on the selection and prediction of academic performances [7], including challenges FTDs face when applying to various IDPs and possible pathways for pursuing dental careers in the United States [2]. However, there is limited evidence on the educational and clinical outcomes of these programs.

FTDs in IDPs have diverse cultural backgrounds. The majority of FTDs who have sought additional training in the U.S. originated from Asia, followed by Eastern Europe, Africa, and Central or South America [6,8]. Previous abroad dental educational experiences may impact the FTDs’ didactic and clinical performance during their matriculation and progression through U.S. dental schools. Non-U.S. dental institutions possess distinct didactic and clinic curricula, as well as educational pedagogies, all of which may impact the fundamental knowledge and clinical performance of FTD students within their IDPs [9]. For instance, some FTDs may have never been exposed to advanced technology during their previous dental training. However, a 2012 study found that when comparing the National Dental Examining Board and Objective Structured Clinical Exam results [10], there was no significant difference between FTDs and traditional students. FTDs seem to be able to transfer previous knowledge, skills, and experiences to progress through IDP programs and acquire the necessary knowledge [6,11].

Data on the clinical experiences of FTDs during their training in IDPs are limited, especially within the context of program length. Dental schools in the U.S. have developed modified IDP curricula ranging from 24 months to 48 months [2,5]. However, the program time allocation may not be evidence-driven. Further outcome assessments are needed on FTDs’ clinical experiences, clinical performance, and written examination results as compared with traditional dental students to help develop a more comprehensive curriculum for FTD students in U.S. dental schools. The objectives of this study were to (1) describe the clinical aspect of the IDP at the University of Illinois Chicago, College of Dentistry (UIC-COD) and (2) compare the clinical experiences of FTDs enrolled in the Advanced Standing DMD Program (DMDAS) versus the traditional DMD program.

### 1.1. Preclinical Curriculum for UIC-COD DMDAS Students

At the UIC-COD, within a span of 28 months, all DMDAS students are provided with robust training to ensure full engagement and a rich experience in comprehensive patient care, extramural programs, enhanced readiness to challenge regional licensure examinations, and time to engage in research and scholarly activity. During the first eight months, the DMDAS students focus on traditional first- and second-year preclinical activities and comprehensive care courses that integrate foundational knowledge and clinical learning. All DMDAS students, regardless of their past dental experience, meet all preclinical course objectives in an accelerated fashion, asynchronous from the traditional DMD students. The course load during the preclinical semesters is heavy, as many competencies are met in preparation for entry into the clinic. At the end of the preclinical training, all DMDAS students acquire skills and knowledge comparable to the DMD students before they are promoted into the clinics.

### 1.2. Clinical Curriculum for UIC-COD Students

At UIC-COD, student competency is assessed based on input from various sources via a holistic modified portfolio, referred to as the UIC-COD Competency Model. Information on students’ performance and progression towards clinical competency is gathered using the following four metrics: (1) Faculty Observation, (2) Self-Evaluations, (3) Capstone Appraisal Procedures (CAPs), and (4) Varied Experiences. Upon the completion of the predoctoral dental program, with the use of the data points acquired from this competency model, the faculty can assess overall competency and predict the graduate’s readiness to practice independently, beginning general dentistry.

**Faculty Observation:** Each student’s patient encounter in the clinic is assessed by a calibrated faculty member daily. The faculty member evaluates the student based on seven clinical domains (Professionalism, Ethical Behavior, Cultural Competence; Dialogue; Assessment; Differential Diagnosis; Treatment Plan; Intervention; and Evaluation of Outcomes) with respect to progression towards clinical competency, and records the evaluation in the electronic health record. Each domain has a defined grading rubric, and all clinical faculty members are calibrated to he assessment using the rubric. At the end of the semester, all faculty members who have worked with a student provide the student a summative grade, with formative feedback in the form of comments.

**Student Self-Evaluation:** A students’ ability to self-evaluate their professional growth consistently, systematically, and accurately is paramount. To this end, the students are provided with training, opportunity, and feedback, to hone those skills throughout their dental education. In patient care, all the performance examinations and independent high stakes examinations have a requirement to pass not only the clinical aspect, but the robust self-evaluation component of the performance examination. Other forms of self-evaluation via portfolio, case report completion at the end of the final year and reflective papers throughout the curriculum have been documented.

**Capstone Appraisal Procedures (CAPs):** UIC-COD defines a CAP as an independent, timed assessment that evaluates the skill of a student in an authentic environment. The assessment of the CAP is carried out by a faculty member using a written referenced-based criteria (rubric), to which the faculty member has been calibrated. At UIC-COD, two types of examinations are concurrent with the above definitions—Objective Structured Clinical Exams and Performance Exams. Students must successfully challenge three separate OSCEs in their clinical years. An OSCE focusing on the behavioral sciences and patient management is administered in the 3rd year. An orthodontic OSCE is given as part of the orthodontic rotation in the 4th year. In 2020, a new 21 station multidisciplinary, clinical OSCE was introduced that students must successfully challenge in the final year. There are over a dozen performance exams in various disciplines (e.g., restorative, periodontics, endodontics, pediatric dentistry, urgent care, oral surgery) that rigorously assess student competency towards beginning independent practice. Students must successfully challenge all PEs prior to graduation along with the self-assessment component of the PE.

**Varied Experiences:** Within the College, patient care is comprehensive and provided with the faculty and patient approval of phased and sequenced treatment plans. The resulting comprehensive care provided to patients by the dental students is most often multidisciplinary in nature. The exposure to and experiences from the various patients seen in the comprehensive clinics results in an excellent comprehensive care experience over the longitudinal course of patient treatment. Currently, the traditional DMD students partake in 6 semesters of patient care and the DMD-AS students partake in 5 semesters of clinical experience. It is typical for a student to have a mature roster of 40–50 active and recall patients at any one time. Students are regularly monitored by faculty to ensure adequate varied experiences. In 2014, the UIC-COD transitioned to relative value units (RVUs) to aid in monitoring student productivity and ensure varied student experiences as detailed above. Each procedure completed in the clinic has an associated relative value unit. An RVU is a numerical value that is given to a clinical procedure based on the time, expertise, and educational experience needed to complete the procedure [12]. All varied clinical experiences are captured daily via institutionally designated RVUs assigned to each procedure. To ensure clinical utilization to maximize the student’s education, the students must obtain a minimal number of total RVUs each semester. Once the minimum is attained, students receive a higher grade based on the number of total RVUs earned each semester. In 2017, a newly developed Minimal Essential Experiences (MEEs) report was created. For each discipline, students are expected to obtain adequate experiences as measured by total RVUs within each category. Minimum RVU thresholds are designated for each discipline based on historical data, student cohort, and year of training; students are expected to surpass these thresholds within a designated timeframe. MEE thresholds are reviewed annually by the clinic directors and adjusted based on the prior year’s statistical data. Additionally, the liaisons in the specialties of endodontics, periodontics, pediatrics, orthodontics, and oral surgery provide input as needed. Having minimal RVU totals per semester, and minimal totals of RVUs in each MEE category for the student to attain, assists with student accountability towards the completion of varied experiences and the optimal utilization of clinical opportunities. RVUs and MEEs are by no means solely used to measure competency, only to ensure that students have the necessary varied experiences to safeguard their progress towards clinical competency.

**Figure 1:**
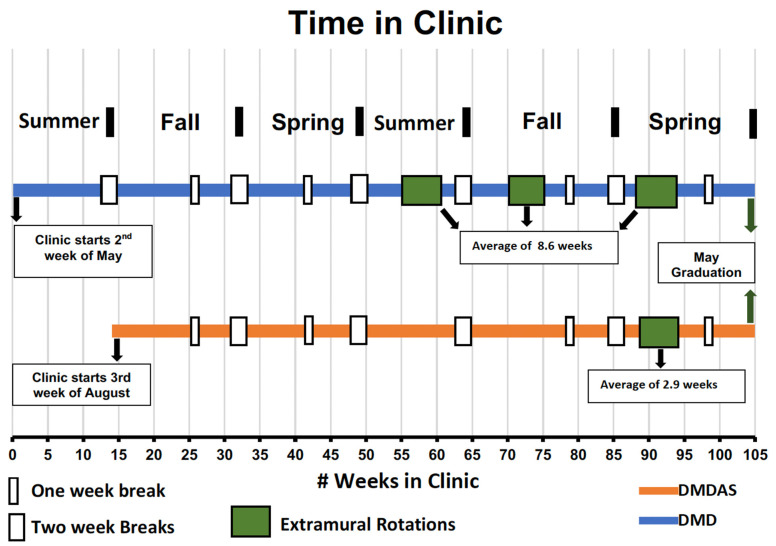
The timeline of DMD and DMDAS students from start of clinic activity to graduation. DMD students’ clinical activities span 24 months (105 weeks total, 77 weeks when breaks and extramural factored) in duration. DMDAS students’ clinical activities span 18 months (90 weeks total, 68 weeks when breaks and extramural factored) in duration. Colored vertical bars during represent the number of weeks students are on extramural sites to obtain experiences in community health settings. For DMD students, this ranges from 4–16 weeks and for DMD-AS students 2–4 weeks (average of 8.6 weeks for DMD and 2.9 weeks for DMDAS students).

Traditional DMD students start their clinical activity at the beginning of the summer semester of their 3rd year, whereas DMDAS students join the clinic at the beginning of the fall semester. A timeline of clinical activities for both cohorts is depicted in Figure 1. Prior to entering clinics, the DMDAS students have had 2 semesters of preclinical and didactic activities. An in-depth analysis of total clinic time indicates that when factoring for extramural rotations, DMD students on average have 77 weeks of available clinical opportunities at the College of Dentistry as compared to 68 weeks for DMDAS students. The MEE and RVU expectations are initially different for the two cohorts as the DMD students have additional time in the clinic as compared to the DMDAS students; however, the expectations converge by the end of the final semester. RVUs and MEEs are only recorded when students obtain clinical experiences within the College of Dentistry. The purpose of the current study was to determine if the clinical experiences of the DMDAS students, as measured by RVUs, was comparable to that of the DMD students within the UIC, College of Dentistry.

## 2. Materials and Methods

This study was exempted by the Institutional Review Board at the University of Illinois Chicago (#2022-0386). A cross-sectional, retrospective chart review of patients cared for by UIC-COD predoctoral dental students using the electronic health record (axiUm, Exan, Coquitlam, BC, Canada) was conducted. The electronic health record (EHR) is defined as an electronic version of patients’ medical records that includes all key administrative clinical data relevant to patient care. The in-house enhanced EHR included student clinical experience tracking mechanisms. Within the EHR system, students’ performance exam records and varied experiences are captured and displayed via a real-time dashboard function. Clinical experience data as measured by RVUs, acquired through entered procedure codes from DMD and DMDAS graduating classes of 2018, 2019, 2020, 2021, and 2022, were retrieved from the electronic health record. Acquired total RVUs were categorized based on various dental disciplines and procedure types (e.g., diagnosis, prevention, direct restorations, indirect restorations, endodontics, periodontics, digital restorations, implant-supported crown, removable prostheses, and oral and maxillofacial surgery). Minimal Essential Experience (MEE) thresholds established based on the historical data were reported. The observation timeframe included each semester after students matriculated in the clinical setting. The number of semesters of the Advanced Standing DMD program (DMDAS) and the traditional DMD program in the clinic were also documented. Any data from students who discontinued their dental education or were placed on probation with education agreements that extended their time at UIC-COD were excluded.

Subject information was extracted by the co-investigators (Z.A., F.S.A., J.C.Y.). Student names and ID numbers were excluded from the data set. RVUs per student for clinical procedures categories as described above were collected and averaged per student in each class and cohort (DMD vs. DMDAS). All data were entered and coded into a spreadsheet (Excel 2016; Microsoft Corp, Redmond, WA, USA). The demographic information of the students among the DMD and DMDAS cohorts studied was collected. Descriptive analyses including the mean, standard deviation, and percentile differences of RVUs in each discipline were calculated and compared between the 2 cohorts. The mean and standard deviation of RVUs in each semester were also computed for both cohorts. A statistical software program (IBM SPSS Statistics, v25.0; IBM Corp, Armock, NY, USA) was used for descriptive and statistical analyses. Students’ clinical procedure experiences at the end of each year were compared (DMD vs. DMD AS) using independent *t*-test (α = 0.05).

## 3. Results

**Figure 2:**
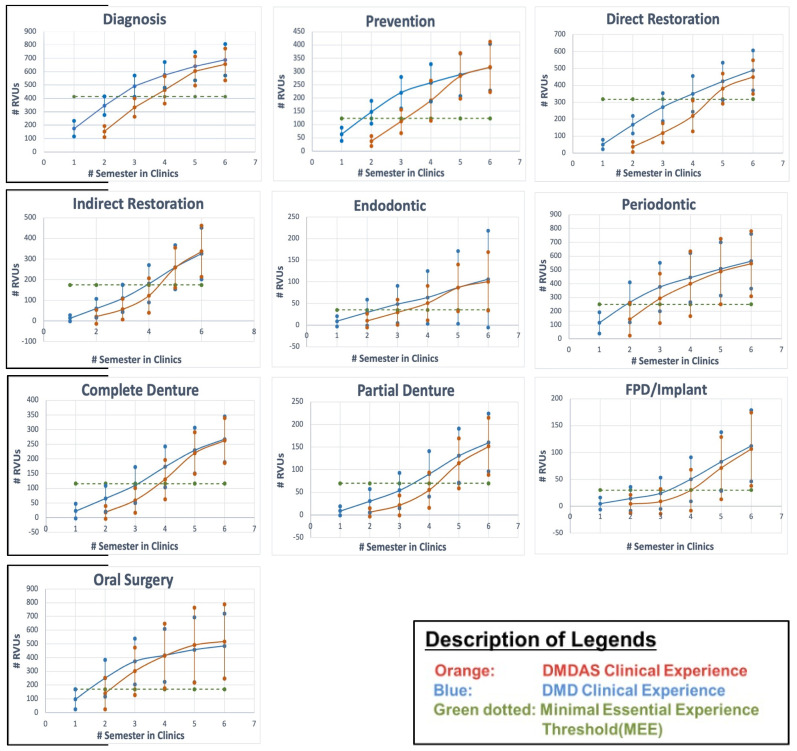
Mean and Standard Deviation RVUs each semester for DMD vs. DMDAS students within various disciplines.

The distribution of the subjects of students among the DMD vs. DMDAS groups within each class is demonstrated in Table 1. The study evaluated 295 DMD and 253 DMDAS students, while excluding 6 DMDAS and 11 DMD students from the data set due to the discontinuation of their dental program or placement on academic probation with an educational agreement that extended their time at the UIC-COD. The results indicated that DMD and DMDAS students have comparable clinical experiences by the end of their final year even though the DMDAS have less time dedicated to the clinics at the UIC-COD (Table 2). On average, DMD students completed slightly more clinical procedures compared with their foreign-trained DMDAS peers in the categories of diagnosis (688 ± 118 RVUs vs. 655 ± 119 RVUs, *p* = 0.002), direct restorations (488 RVUs ± 118 vs. 449 ± 100 RVUs, *p* < 0.001), periodontics (563 ± 198 RVUs vs. 545 ± 236 RVUs, *p* = 0.328), and removable partial dentures (160 ± 64 RVUs vs. 152 ± 63 RVUs, *p* = 0.137). Clinical experiences in implants/fixed partial dentures (112 ± 66 RVUs vs. 106 ± 68 RVUs, *p* = 0.305), prevention (316 ± 88 RVUs vs. 317 ± 95 RVUs, *p* = 0.919), complete dentures (267 ± 78 RVUs to 263 ± 77 RVUs, *p* = 0.506), and endodontics (106 ± 112 RVUS vs. 101 ± 68 RVUs, *p* = 0.492) were comparable. DMDAS students obtained more experiences in the categories of indirect restorations (337 ± 124 RVUs vs. 326 ± 125 RVUs, *p* = 0.276) and oral surgery (518 ± 170 RVUs vs. 484 ± 273 RVUs, *p* = 0.150). In all categories, MEE thresholds were surpassed by both cohorts (Figure 2).

## 4. Discussion

Future projections for the demand in dentistry are complex and often driven by underlying factors impacting both supply and demand [13]. However, the future need for dentists in the U.S. is apparent as the size of the population continues to rise, estimated to increase to 61.5 million people in 2040 [13], while a large group of dentists are entering the retirement phase [14]. These needs can potentially be fulfilled by FTD graduates from accredited dental institutions in the United States as they enter the workforce more rapidly because of the shorter duration of IDPs. Furthermore, improvements in workforce diversity in health professions such as dentistry are critical not just from a social justice point of view; moreover, to help improve access, equity, and the quality of care [15,16,17]. Projections estimate that by 2044, more than half of the U.S. is predicted to be from a minority group. Minority patients are more likely to seek care from a provider with a similar race or ethnicity, thus helping improve health disparities among the population [18,19,20,21,22]. Foreign-trained dentists may be essential in meeting the current and future needs of the U.S. population while bringing diverse perspectives, values, and understanding along with multilingual skills to the health care profession [3,22].

The DMDAS program at the UIC-COD almost has the same numbers of cohorts as the regular DMD, 50 vs. 50–70, respectively, which may contribute to the validity of the study. Further, the DMDAS program consists of a 7 semester (28 months) curriculum—2 semesters of preclinical care (simulation and review of biomedical sciences) designed specifically for the DMDAS students and 5 semesters of clinical patient care. During the clinical patient care component, the DMDAS students track with the DMD students, who partake in 6 semesters of clinical opportunities. On average, DMDAS students have 9 weeks fewer clinical opportunities at the College of Dentistry as compared with their counterparts. The results of this study indicated that at the UIC-COD, foreign-trained dental students obtain comparable clinical experiences to those of the traditional DMD students, as measured by relative value units. Although the differences in diagnosis and direct restorations reached statistical significance, the clinical impact may not be significant, as both cohorts surpassed the Minimal Essential Experience thresholds. Further exploration may be warranted to investigate the underlying reason for the observed trend.

Unlike traditional dental programs, the curricular time allocated to dental programs for FTDs is not consistent among U.S. dental schools. Based on each institution’s goals and mission, the dental curriculum for IDPs ranges from 24 to 48 months, averaging around 28 months [1,5]. Each IDP is uniquely developed within the constraints of their corresponding institution to ensure that FTDs are provided with an optimal education and clinical experience to become competent entry level general dentists as mandated by the Commission of Dental Accreditation. To the best of the authors’ knowledge, no study has been published evaluating the length of time for international dental programs nor the students’ overall clinical performance. In the medical literature, multiple studies compared the performance of international medical graduates (IMGs) to U.S. medical graduates (USMGs) during their residency programs [23,24]. A study comparing diagnostic radiology and intervention radiology residency programs indicated that IMGs performed about the same as USMGs [23]. Another study comparing the research and academic productivity of the IMG and USMG plastic surgeons concluded that both cohorts have nearly equivalent research and academic productivity [24]. A study of peer and faculty perceptions on the performance of IMGs in pediatric residency reveals that IMGs performed nearly equal to their USMG counterparts [25].

How are FTDs able to complete similar clinical expectations when compared with the traditional dental students in a shorter time span? FTDs have been reported to be older and have families; they differ from traditional dental students in not only cultural differences but also previous professional and personal life experiences [26,27,28]. On the contrary, the current health professional student body is a more homogenous group, composed largely of Generation Z, or iGens, born between 1995 and 2012 [29,30,31]. Since birth, iGens have been shaped by the internet, the use of ubiquitous technology such as smartphones, and the all-encompassing impacts of social media [29]. iGen members tend to develop life skills at a slower pace, with reports indicating that many at the age of 18 resemble 14 year-olds of the previous generation in terms of social and life skills [29]. This is most likely due to the decreased responsibilities and opportunities for independence during their formative years. They display characteristics that include cautiousness and pragmatism, poor face-to-face social interaction skills, and difficulty with time management or balancing multiple responsibilities concurrently [29,32]. These distinct characteristics of iGens may be impediments to optimal performance within a health profession; thus, traditional professional students may have a steeper learning curve with respect to time. As stated earlier, FTDs tend to be older and more heterogeneous, with less “iGen characteristics”. These differences may have in some shape or form led to the observed results noted in the current study.

To be able to develop a consistent program for foreign-trained dentists among dental schools in the United States, each dental school needs to review their international dental program and compare data-driven outcomes to their traditional dental program. While this study provides the readership with valuable information about the quantity of the clinical experiences of the FTDs compared with the traditional dental students in one institution, limitations include a lack of information about the quality and comprehensive understanding of their clinical experiences. A numerical account of experiences does not directly translate to clinical competency but, as outlined in the introduction, is one aspect of the UIC-COD Competency Model. Furthermore, correlation does not translate to causation. Similar results may be observed in other institutions with dissimilar teaching pedagogies, curriculum, and program length. Students may work at different paces knowing the length of the program, as may the faculty/staff in charge of disseminating patient cases, to ensure successful timely graduation of all students. Future studies are needed to determine the quality of student performance, critical thinking skills, and an in-depth knowledge of clinical dentistry among DMDAS and DMD students. Studies assessing student perceptions of their clinical experiences using a mixed-method approach can also enhance further understanding of IDPs.

## 5. Conclusions

This paper provides a potential framework for the self-assessment of current and/or future International Dental Programs based on each institution’s individual goals, values, and competency models. A 28 month program for foreign-trained dentists appeared to be a reasonable timeframe for students to obtain the adequate number of varied clinical experiences as compared with a traditional four-year program in the University of Illinois Chicago, College of Dentistry. The results indicated both cohorts surpassed program-established Minimal Essential Experience thresholds in all disciplines.

## Figures and Tables

**Table 1:** Distribution of DMD and DMDAS students among the various classes included in the study.

Student Demographics per Class and Cohort								
	DMD				DMDAS			
	Number	Gender (Female)	Gender (Male)	Average Age (Years)	Number	Gender (Female)	Gender (Male)	Average Age (Years)
Class of 2018	48	22	26	27	52	31	21	33
Class of 2019	49	23	26	28	50	31	19	34
Class of 2020	66	38	28	27	48	35	13	35
Class of 2021	66	27	39	27	52	38	14	35
Class of 2022	66	37	29	27	51	35	16	35
Total	295	147	148	27	253	170	83	34

**Table 2:** Average total RVUs per category for DMDAS vs. DMD students.

Average RVUs per Category, Comparing DMDAS and DMD Cohorts’ Class of 2018–2022				
Disciplines	DMDAS (Mean (SD))	DMD (Mean (SD))	Percentile Differences	*p* Value
Diagnosis	655 (119)	688 (118)	−5%	0.002
Prevention	317 (95)	316 (88)	0%	0.919
Direct Restoration	449 (100)	488 (118)	−8%	<0.001
Indirect Restoration	337 (124)	326 (125)	3%	0.276
Endodontics	101 (68)	106 (112)	−5%	0.492
Periodontics	545 (236)	563 (198)	−3%	0.328
Complete Dentures	263 (77)	267 (78)	−2%	0.507
Partial Dentures	152 (63)	160 (64)	−5%	0.137
Implant/FPD	106 (68)	112 (66)	−6%	0.305
Oral Surgery	518 (170)	484 (273)	7%	0.15

## Data Availability

The raw data supporting the conclusions of this article will be made available by the authors on request.
